# The Effects of *Buthotus schach* Scorpion Venom on Electrophysiological Properties of Magnocellular Neurons of Rat Supraoptic Nucleus

**Published:** 2018

**Authors:** Akram Aboutorabi, Nima Naderi, Hamid Gholami pourbadie, Hossein Zolfagharian, Hossein Vatanpour

**Affiliations:** a *Student Research Committee, School of Pharmacy, Shahid Beheshti University of Medical Sciences, Tehran, Iran.*; b *Department of Toxicology and Pharmacology, School of Pharmacy, Shahid Beheshti University of Medical Sciences, Tehran, Iran.*; c *Department of physiology and pharmacology, Pasteur Institute of Iran, Tehran, Iran.*; d *Department of venomous animals and antivenom production Razi vaccine and serum research institute Agricultural research education and extension organization ( AREEO), Karaj, Iran.*

**Keywords:** *Buthotus schach*, Ion channels, Whole-cell patch clamp, Scorpion venom

## Abstract

*Bothutous Schach* (BS) scorpion venom consists of several polypeptides that could modulate ion channels. In this study, the effects of BS crude venom on passive and active electrophysiological properties of rat neurons in supraoptic nucleus (SON) of hypothalamus was investigated using whole-cell patch clamp technique. The results showed that bath application of BS venom produced significant change in passive properties of SON neurons, namely a decrease in resting membrane potential and an increase in input resistance of the cells. Also, significant change in active properties of SON neurons was shown after bath application of BS venom; including a decrease in the number of evoked action potential along with an increase in half width and decay time of action potential and a significant decrease in after-hyperpolarization amplitude. Finally, a decreased latency to the first spike accompanied by a lower current threshold to elicit the first spike was shown compared with the values before venom application. These effects are possibly through blocking different ion channels including potassium channels. Further experiments using different fractions of the venom is required to specify venom effects on various ion channels.

## Introduction

Approximately 1500 various species of scorpions divided into thirteen families are reported throughout the world (1); from which Buthidae family is considered to be the largest and the most medically important (2). The scorpion venom possesses a heterogeneous collection of pharmacologically active polypeptides such as neurotoxins, antimicrobial peptide, proteases, and cytolytic peptides; some of them may serve as leading compounds for drug design with therapeutic value (3, 4). In excitable and non-excitable cells, the scorpion neurotoxins block or modify ion channel functions (4). The scorpion venoms are generally classiﬁed into major groups of neurotoxins. The first group is formed by short-chain peptides, consist of 20 to 43 amino acid residues and usually act as potassium channels blocker. The other group with long-chain peptides scorpion toxins, containing 58 to 76 amino acid residues is modiﬁer of the sodium channels (5). In excitable cells, it is well accepted that pharmacological agents interacting with ion channels signiﬁcantly change the physiological properties of the cells. After development of cellular electrophysiology, the physiological roles of different ion channels were discovered in a variety of cells (6). *Buthotus Schach* (BS) or *hottentotta zagrosensis* is known as one of the most dangerous scorpions, with limited distributed species of scorpion from Buthidae family in the middle west of Iran (7). BS venom can cause convulsion, arrhythmia, respiratory depression, and cardiac arrest in human. Previous studies indicated that BS venoms can cause paralytic effects on nerve-muscle preparations (8, 9). The purpose of this study was to investigate the effects of BS scorpion crude venom on intrinsic and active electrophysiological properties of the magnocellular neurons (MCNs) of supraoptic nucleus (SON) in rat hypothalamus, using patch-clamp techniques. 

## Experimental

*Venom*

Crude BS venom was provided by the Department of Poisonous Animals, Razi Vaccine and Serum Research Institute, Karaj, Iran. The crude venom was obtained by electrically stimulating the telson of scorpions and then was lyophilized (10). 

*Animals*

Male wistar rats (60–80 g) were obtained from Pasteur Institute (Tehran, Iran), were allowed free access to water and the pellet diet under standardized housing conditions with a 12 h:12 h light:dark cycle and at 22 ± 2 ºC temperature with a relative humidity of 40%. All experiments were carried out according to the guidelines of the National Institutes of Health (NIH Publications No. 80-23, revised 1996), and approved by the Ethics Committee at Shahid Beheshti University of Medical Sciences.

*Preparation of slices*

Rats were anesthetized with isoflorane, the brain was immediately removed and placed in ice-cold slicing solution containing (in mM): 206 sucrose, 2.8 KCl, 1 MgCl_2_, 2 MgSO_4_, 1 CaCl_2_, 1.25 NaH_2_PO_4_, 26 NaHCO_3,_ and 10 D-glucose and was continuously bubbled with O_2_ (95%) and CO_2_ (5%). Coronal slices containing hypothalamic region were prepared using a vibrating microtome (Campden Instruments, UK). The Slices were then transferred to an incubation chamber, where they were maintained submerged at 32-34 °C for one hour in a CSF solution (described later) and then kept at room temperature until time of recording. 

*Whole cell patch-clamp recordings*

The slices were transferred to recording chamber set on the stage of an upright microscope (Olympus BX51W1, Japan). Patch clamp recording was done on MCNs of SON that were identified based on method described by Hirasawa *et al.* (11). The slices were perfused by artificial cerebrospinal fluid (aCSF) solution containing (in mM) 124 NaCl, 2.8 KCl, 2 CaCl_2_, 2 MgSO_4_, 1.25 NaH_2_PO_4_, 26 NaHCO_3_, and 10 D-glucose (Osmolarity = 290 mOsm) bubbled with a mixture of O_2_ (95%) and CO_2_ (5%) at room temperature (25 ± 2 ºC). Whole cell recordings were done under visual control using infrared difference interference contrast (IR-DIC) optics (Hamamatsu, Japan). Whole cell recordings were made using Multiclamp 700B amplifier (Axon Instruments, USA) equipped with Digidata 1320 A/D converter (Axon Instruments, USA). Recordings were made using borosilicate glass pipettes (1.2 mm O.D., 0.9 mm I.D.) with 4–7 MΩ resistance when they were filled with intracellular solution consisting (in mM): 90 potassium gluconate, 30 KCl, 2 MgCl_2_, 2 EGTA, 5 NaCl, 10 HEPES (pH = 7.25; osmolarity = 290 mOsm). Recordings were accepted if the series resistance was less than 25 MΩ, and if it did not vary by 20% during the experiment.

After establishing the whole-cell recording configuration in current-clamp condition, the cell was permitted to stabilize for 1–2 min to allow equilibration between the cell interior and the micropipette solution. The resting membrane potential was then measured. The membrane input resistance (R_in_) was determined by applying 1000 ms hyperpolarizing current pulses (0 to -200 pA; -50 pA increment) and calculating the slope of the resultant current-voltage (I-V) curve within the linear portion. The action potential (AP) half-width was measured at one half of spike maximum amplitude. The difference between the AP threshold and the minimum voltage following the AP spike was determined as the after-hyperpolarization (AHP) amplitude. Moreover, to determine the variation in neuronal excitability and the evoked firing properties, trains of action potentials were induced by injecting depolarizing current (50 to 250 pA, 50 pA increment, 1000 ms from a holding potential of -60 mV) and number of action potentials as well as the onset half-width, and fast action potential after-hyperpolarization (AHP) amplitude were measured in each depolarizing current phases. Finally, baseline firing was recorded by injecting ramp currents (200 pA, 1000 ms) into SON to mimic the small and slow depolarization of membranes. After SON reached an equivalent baseline level of firing, the effect of venom on neuron excitability was measured. 

*Statistical Analysis*

All analyses were performed using pClamp® software, version 10.5 (Molecular device Inc.). Further statistical analyses were performed using the Graph pad Prism, version 6 (Graph Pad Software Inc.). Two-way analysis of variance (ANOVA) followed by Bonferroni’s post-test was carried out to analyze differences between groups. *p*-value of less than 0.05 was considered as statistically significant. All data were presented as mean ± SEM.

## Results

*Effect of BS venom on passive properties of SON neurons*

**Figure.1:**
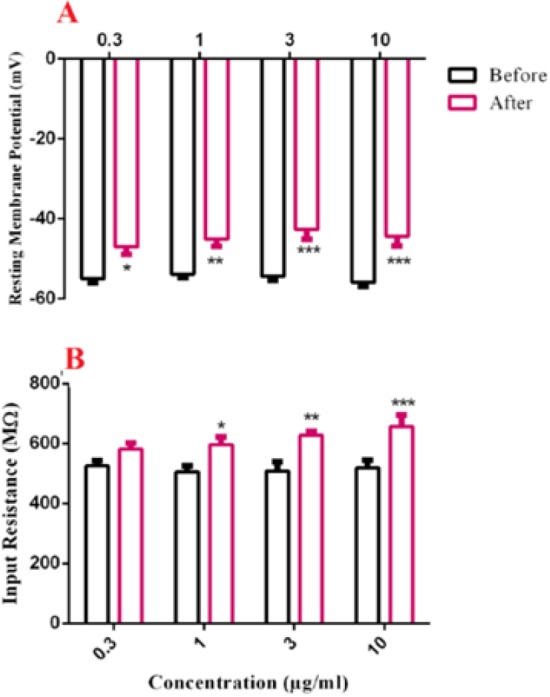
Effects of bath application of BS scorpion venom [0.3, 1, 3 and 10 µg / mL] on resting membrane potential (A) and input resistance (B) of SON neurons. Data are shown as mean ± SEM. *P < 0.05, **p < 0.01 and ***p < 0.001 significant difference compared with the values before venom application

As shown in Figure. 1A, bath application of various concentrations of BS venom produced significant decrease in resting membrane potential of SON neurons [F (1, 33) = 61.24, *p *<0.0001]. Further analysis using Bonferroni’s post test revealed significant decrease in resting membrane potential after bath application of venom 0.3 µg/mL (*p *<0.05), 1 µg/mL (*p *<0.01), 3 and 10 µg/mL (*p *<0.001). Also, bath application of BS venom caused significant change in R_in_ of SON neurons [F (1, 32) = 39.39, *p *<0.0001; Figure. 1B]. Further analysis by Bonferroni’s test revealed a significant increase in R_in _after bath application of venom at 1 µg/mL (*p*<0.05), 3 µg/mL (*p*<0.01), and 10 µg/mL (*p*<0.001) compared with the values before venom application.

*Effect of BS venom on action potential properties of SON neurons*

**Figure 2.**
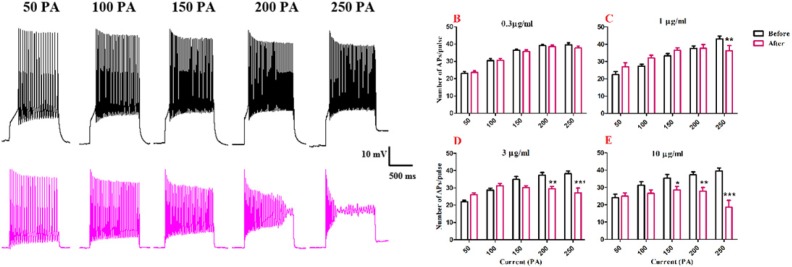
Effect of bath application of BS venom on evoked action potentials by depolarizing currents. (A) Sample traces representing changes in the number of evoked action potentials per pulse before and after bath application of BS venom. Changes in the number of evoked action potentials per pulses were assessed before and after bath application of venom at 0.3 (B), 1 (C), 3 (D), and 10 µg/mL (E). Data were shown as mean ± SEM (N = 6-8 cells). *p <0.05, **p <0.01, ***p <0.001 and ****p <0.0001 compared with the values before bath application of BS venom

To evaluate the effects of BS venom on the excitability of SON cells, trains of action potentials were evoked in the cells by applying depolarizing current pulses (1000 ms duration) ranging from 50 to 250 pA at holding potential of -60 mV. Traces were shown in Figure. 2A. Firing rates were significantly decreased after bath application of venom at 1[F (1, 25) = 2.342, *p* <0.0001; Figure. 2C] 3 [F (1, 25) = 11.55, *p *<0.0001; Figure. 2D] and 10 µg/mL [F (1, 25) = 70.37, *p *<0.0001; Figure. 2E]. Further analysis showed that after bath application of venom (1 µg/ml) significantly decreased in the number of action potential at 250 pA (*p*<0.01), also after bath application of venom (3 µg/ml), a significantly decrease in the number of action potentials per pulse at 200 pA (*p*<0.01) and 250 pA (*p*<0.001) were observed compared with the values before venom application. Also, bath application of venom (10 µg/mL) decreased the number of action potential at 150 pA (*p*<0.05), 200 pA (*p*<0.01,) and 250 pA (*p*<0.001) compared with the baseline values.

**Figure 3:**
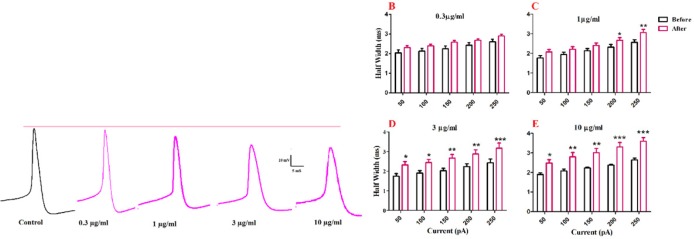
Effect of bath application of BS venom on evoked action potentials by depolarizing currents. (A) Sample traces representing changes in the half- width and AHP amplitude of evoked action potentials per pulse before and after bath application of BS venom. Changes in AHP amplitude of evoked action potentials per pulses were assessed before and after bath application of venom at 0.3 (B), 1 (C), 3 (D), and 10 µg/mL (E). Data were shown as mean ± SEM (N = 6-8 cells). *p <0.05, **p <0.01, ***p <0.001 and ****p <0.0001 compared with the values before bath application of BS venom

**Figure. 4:**
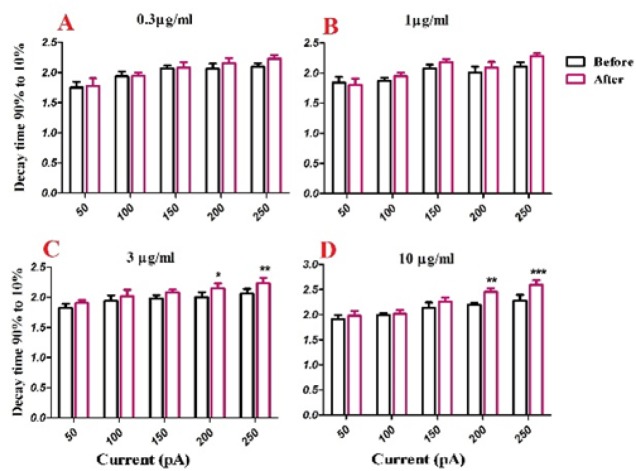
Effect of bath application of BS venom on evoked action potentials by depolarizing currents. Changes in Decay time of evoked action potentials per pulses were assessed before and after bath application of venom at 0.3 (A), 1 (B), 3 (C), and 10 µg/mL (D). Data were shown as mean ± SEM (N = 5-8 cells). *p <0.05, **p <0.01 and ***p <0.001 compared with the values before bath application of BS venom

Half width was significantly increased after bath application of venom at 1 µg/mL [F (1, 30) = 40.43, *p *< 0.0001; Figure. 3C], 3 µg/mL [F (1, 25) = 66.73, *p *< 0.0001; Figure. 3D], and 10 µg/mL [F (1, 25) = 81.45, *p *< 0.0001; Figure. 3E] concentrations. Further analysis revealed a significant increase in the second and third half-width of APs after bath application of venom (1µg/mL) at 200 pA (*p *< 0.05), and 250 pA (*p *< 0.01) depolarizing current injections. Moreover, bath application of venom (3µg/mL) increased half-width of APs at 50 pA (*p *< 0.05), 100 pA (*p *< 0.05), 150 pA (*p *< 0.01), 200 pA (*p *<0.01), and 250 pA (*p *< 0.001) current injections. Finally, BS venom (10 µg/mL) increased the half-width of APs at 50 pA (*p* < 0.05), 100 pA (*p *< 0.01), 150 pA (*p *< 0.01), 200 pA (*p *< 0.001), and 250 pA (*p *< 0.001) current injections. The observed increase in half-width was in part due to an increase in decay time of action potential, as shown in Figure. 4. Bath application of BS venom caused a significant change in decay time at 3 µg/mL [(1, 20) = 31.93, *p *< 0.001; Figure. 4D], and 10 µg/mL [(1, 20) = 27.45, *p *< 0.001; Figure. 4E] concentrations. Further analysis revealed a significant increase in decay time after bath application of venom at 3 µg/mL at 200 pA (*p* < 0.05), and 250 pA (*p *< 0.01) depolarizing current injections. Moreover, bath application of venom (10 µg/mL) increased decay time of APs at 200 pA (*p *< 0.01), and 250 pA (*p *< 0.001) current injections.

**Figure 5:**
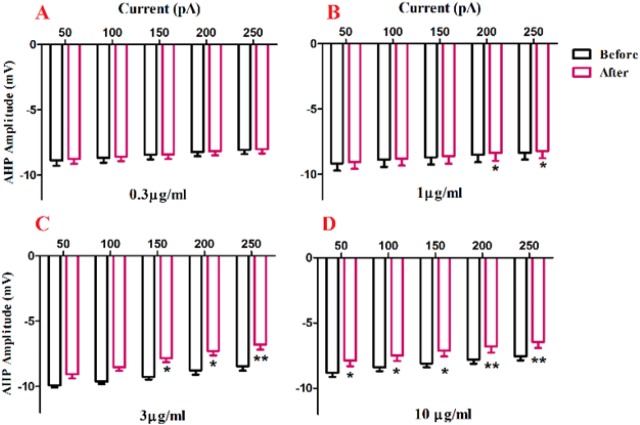
Effect of bath application of BS venom on evoked action potentials by depolarizing currents. Changes in AHP amplitude of evoked action potentials per pulses were assessed before and after bath application of venom at 0.3 (A), 1 (B), 3 (C), and 10 µg/mL (D). Data were shown as mean ± SEM (N = 6-8 cells). *p <0.05, **p <0.01, ***p <0.001 and ****p <0.0001 compared with the values before bath application of BS venom

A significant change in AHP amplitude of action potential was shown after bath application of venom at 1 µg/ml [F (1, 20) = 41.83, *p* <0.0001; Figure. 5B], 3 µg/mL [F (1, 25) = 43.57, *p *< 0.0001; Figure. 5C], and 10 µg/mL [F (1, 25) = 58.80, *p *< 0.0001; Figure. 5D] concentrations. Further analysis revealed a significant decrease in AHP amplitude of action potential after venom application compared with the values before venom application. The observed significantly decrease in AHP amplitude of action potential after application of 1 µg/mL of venom at in 200 pA (*p *< 0.05) and 250 pA(*p *< 0.05), and in 1 µg/mL of venom at 150 pA (*p *< 0.5) 200 pA (*p *< 0.05) 250 pA (*p *< 0.01) and 10 µg/mL at 50 pA (*p *< 0.5) and 100 pA (*p *< 0.5) 150 pA (*p *< 0.5) 200 pA (*p *< 0.01) 250 pA (*p *< 0.01).

**Figure.6.**
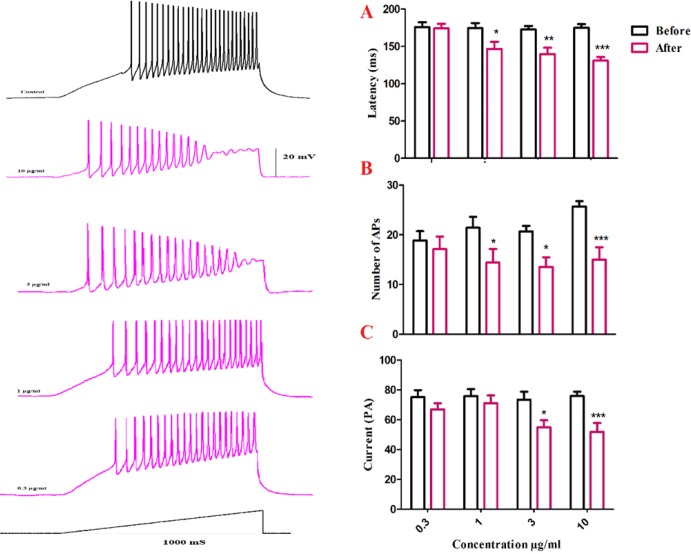
Changes in firing properties of SON cells in response to ramp currents. (A) Representative spike firings of SON cells in response to ramp current clamp before and after venom application. Bath application of venom induced significant decrease in latency of the first AP (B), a significant decrease of current required to evoke the first AP (C), and a significant decrease in AP numbers (D). Data are shown as mean ± SEM. *p <0.05, **p <0.01, ***p <0.001 significant difference compared with the values before venom application

To further evaluate BS venom effects on evoked action potentials of SON neurons, the latency to the first spike elicited by depolarizing currents was measured before and after bath application of BS venom. As shown in Figure. 5B, bath application of venom produced significant change in the latency of the first evoked action potential [F (1, 24) = 34.05, *p *<0.001; Figure. 6B]. Post-hoc analysis revealed a significant decrease in the latency after bath application of the venom at 1 (*p *< 0.05), 3 (*p *< 0.01), and 10 µg/mL (*p *< 0.001) compared with the values before venom application. The decreased latency to the first spike was accompanied by a lower current threshold to elicit the first spike compared with the values before venom application [F (1, 22) = 27.04, *p *< 0.001; Figure. 6C]; which was significantly different at 3 (*p *< 0.05) and 10 µg/mL (*p *< 0.001). Moreover, bath application of venom produced significant change in the number of action potentials [F (1, 24) = 33.40, *p *< 0.001; Figure. 6D]. Post-hoc analysis revealed a significant decrease in the latency after bath application of the venom at 1 (*p *< 0.05), 3 (*p *< 0.05), and 10 µg/mL (*p *< 0.001) compared with the values before venom application.

## Discussion

Scorpion toxins contain peptides that target, and usually block ion channels of excitable membranes, including sodium, potassium, and chloride channels. Previous studies in our lab reported inhibitory effect of BS crude venom on neuromuscular transmission, with an initial increase in acetyl choline release followed by blockage of nicotinic receptors (8). Also in another study in our lab observed blocked effect of BS crude venom and its fractions on sodium channels (12). However, the effects of BS crude venom on potassium channels is not clear. In this study, we focused on potassium channels; which are involved in active and passive properties of SON neurons in hypothalamus in current clamp condition. A change in passive properties of SON neurons was shown after bath application of higher concentrations of BS venom, including input resistance and resting membrane potential. Since resting membrane potential and input resistance are largely dependent on the activities of potassium channels (13-16), it could be suggested that the observed decrease in resting membrane potential could be at least partly due to potassium channel. The same results were shown by the putative potassium channel blocker tetraethyl ammonium (TEA), where a significant depolarization in resting membrane potential after bath application of TEA was observed (17). 

Previous reports also demonstrated effects of *Quinquestriatus* toxin (QTX) isolated from the venom of *Leiurus quinquestriatus* (buthidae family) on resting membrane potential of skeletal muscles. It has been shown that application of QTX caused depolarization in resting membrane potential from -82mV to -55mV (18). Similar effects on resting membrane potential and input resistance of hippocampal neurons were shown by Chilean spider venom, with possible contribution of potassium channels (19). 

In order to evaluate the effects of BS venom on active membrane properties, current clamp recordings were performed and changes in the APs properties of SON neurons were investigated. The increase in half-width of APs after venom application, as shown in our results, could suggest inhibitory effects of BS venom on voltage-gated sodium and potassium channels. The increase in half-width and decay time along with a decrease in fAHP amplitude could suggest the possible inhibitory effect of venom on calcium-activated potassium channels. Consistent with our results, bath application of putative BK channel blockers, iberiotoxin (IBTX), and paxillin caused an increase in half-width and a decrease in fast AHP amplitude of spikes in brainstem neurons (20-22). It should be noted that the depolarization of membrane potential could affect the shape of action potential through blockade of voltage gated sodium and potassium channels, thus give rise to changes in firing rate and half-width of action potentials. After BS venom-induced membrane depolarization, inactivation of voltage gated sodium channels would occur that eventually decreases firing rate. Similar situation was shown when potassium channel blocker such as cesium chloride or tetraethyl ammonium was added to extracellular solution (23).

In this study, firing properties of SON neurons evoked by current injections was evaluated to determine the effects of BS venom on voltage gated channels. Changes in the repetitive firing properties in SON neurons were detected during a 1s-duration, 250 pA current injections, including a decrease in the number of AP spikes and AP amplitude during current injection with decreasing trend in voltage amplitudes that eventually resulted in failure of action potential generation by the end of the current step. The inhibitory effect of venom on firing properties of evoked SON neurons suggests an inhibitory effect on voltage gated sodium and potassium channels. In this regard, it has been shown that genetic mutation in Kv2.1 voltage gated potassium channels decreased the repetitive firing properties in primary cortical neurons (21). Such an effect on evoked firing activity was also shown in hippocampal interneurons from NaV1.1 mutant mice (24). The decrease in firing rate could also be related to inhibition of BK potassium channels. Such a decrease in the number and amplitude of APs was also shown in dentate gyrus hippocampal neurons of epileptic rats during a 250 pA current injection, when the cells were treated with BK channel blockers paxillin and iberiotoxin (25). The effect of BS venom on response of SON neurons to slow depolarization was evaluated by injection of ramp currents. Bath application of BS venom decreased latency to first spike and number of spikes. This effect could be due to the blockade of voltage gated sodium channels as proviusly describe by Gu *et al.*, 2007; Shruti *et al*.,(2008). On the other hand venom induced inhibition of A-type potassium channels could theoretically decrease latency to first action potential and also could decrease the number of action potential in RAMP protocol because of an increase in intracellular potassium, shifting the membrane potential to more depolarized potential. In a depolarized neuron, the probability of voltage gated sodium channels inactivated state will increase and could lead to elimination in firing of the cell. The role of A-type potassium channels in latency to first action potential was previously described (13, 26-28), suggesting the important role of this channels in modulation of neuronal excitability (29, 30). 

However, in our study, the effect of BS venom on A-type potassium current was not evaluated and further experiments is required to clarify the effects of BS venom on I_A _currents. There is a discrepancy between occurrence of first AP and number of APs after BS venom application. As we found here, BS venom increased the input resistance. Cells with higher input resistance reach to the AP threshold by applying less amount of current which could be interpreted that they have lower threshold. On the other hand, as demonstrated on the traces, the decreased number of APs is because of failure to AP generation which might be due to use dependently inactivation of voltage gated sodium channels by BS venom. Another explanation for this discrepancy is that the venom may block some leak potassium channels resulting in depolarization of the cells. Cells with depolarized membrane potential have lower threshold and at the same time may produce lower APs due to inactivation of Na channels in the depolarized condition. Such a phenomenon is seen when an excitable cell is bathed with a solution containing high potassium concentration. In this condition, the cell consequently shows lower threshold, depolarized resting membrane potential, and attenuated AP firing. In our work, considering Nernst Equation, blockade of leak potassium channels by BS venom could maintain the cells in a depolarized condition in which voltage gated sodium channels remain in inactivation thus diminish firing rate, and lead to termination of action potentials (23).

## Conclusion

Study on venoms of poisonous animals that are endemic to a specific region of the world provides information regarding the use of these venoms as a pharmacological tool that acts on specific ion channel or ionotropic receptor. Our findings provide electrophysiological evidence demonstrating BS venom application changing both intrinsic and active properties of SON neurons possibly by affecting different types of leaky as well as voltage gated potassium channels. These findings suggest that BS venom could be considered as a pharmacologic tool that allows better understanding of the excitation mechanism of neurons. Additional experiments using various fractions of the venom are needed to evaluate the specific effects of different components of the venom on ion channels subtypes. Also, the effects of BS venom/toxins on different subtypes of potassium channel could be evaluated using single channel recording. Furthermore, use of BS venom, or its specific components, in controlled low doses to modulate of potassium channels, could provide potential therapeutic approaches to control diseases of excitable membranes such as seizure.
